# Advisor-advisee relationship and the organizational culture of doctoral programs on doctoral students’ mental health and academic performance: A scoping review protocol

**DOI:** 10.1016/j.mex.2025.103433

**Published:** 2025-06-11

**Authors:** Beatriz Cintra Storti, Jorge Sinval, Yasmin Lynda Munro, Francisco J. Medina, Marina Greghi Sticca

**Affiliations:** a Faculty of Philosophy, Sciences and Letters at Ribeirão Preto, University of São Paulo, Brazil; b National Institute of Education, Nanyang Technological University, Singapore; c Escola Paulista de Medicina, Universidade Federal de São Paulo, Brazil; d Business Research Unit (BRU-IUL), Instituto Universitário de Lisboa (Iscte-IUL), Portugal; e Faculdade de Medicina, Universidade de Lisboa, Portugal; f Lee Kong Chian School of Medicine, Nanyang Technological University, Singapore; g Facultad de Psicología, Universidad de Sevilla, Spain

**Keywords:** Academic performance, Advisor-advisee relationship, Doctoral programmes, Doctoral students, Mental health, Scoping Review Protocol

## Abstract

Doctoral students’ mental health is increasingly recognized as a critical issue in academia, with advisor-advisee relationships playing a key role in both well-being and academic performance. The organizational culture of doctoral programs may also influence these outcomes, but existing literature has yet to fully addressed the interplay between these factors. This scoping review aims to identify elements within the advisor-advisee relationship and supervision process that are associated with doctoral students’ mental health and academic performance. It also seeks to examine how the organizational culture of doctoral programs relates to these dynamics. The review will include both empirical studies and literature reviews focusing on these relationships. The following databases will be searched: Medline (Ovid), Embase (Ovid), Cochrane Library, Web of Science, ERIC, PubMed, CINAHL (EBSCOhost), and PsycInfo (APA). Studies will be screened by two independent researchers, with duplicates removed. There will be no restrictions on publication date or language. Data extraction will be conducted using a standardized spreadsheet, and findings will be synthesized using thematic analysis, with results presented in both narrative form and summary tables.

## Graphical abstract

**Figure:**
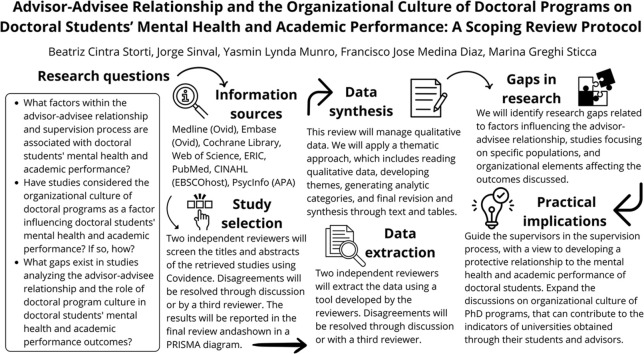

Specifications table

**Table:** 

Subject area:	Psychology
More specific subject area:	*Work and Organizational Psychology*
Name of your protocol:	*Scoping Review Protocol*
Reagents/tools:	*Not applicable.*
Experimental design:	*Scoping review protocol*
Trial registration:	*Open Science Framework*https://osf.io/4w3gd/
Ethics:	*This study meets the criteria of item VI of Article 1 of Resolution 510/2016 of the National Health Council (Ministry of Health, Brazil), which classifies it as a “research conducted exclusively with scientific texts for a literature review.” For this reason, and in accordance with this resolution, the study is neither registered nor evaluated by the CEP (Research Ethics Committees)/CONEP (National Research Ethics Commission) system.*
Value of the Protocol:	*1. Identify the various variables involved in the supervision process of doctoral students, broadening the understanding of how they relate to mental health outcomes and students' academic performance;* *2. Guide the exercise of supervisors in relation to the supervision process of doctoral students, with a view to developing a relationship that is protective to the mental health and academic performance of students;* *3. Expand the space for discussions on organizational culture of graduate programs, represented by policies, standards and guidelines that can contribute to the indicators of universities obtained through their students and advisors.*

## Background

The mental health of doctoral students has emerged as a relevant topic in the academic context, given the concerning statistics that now classify it as a public health issue [1]. The intense demands of studying and the pressure to publish during doctoral training have been linked to mental health problems in this population. Understanding and documenting these challenges is crucial, not only because of the individual needs of these students, but also because such findings have broader implications for the institutions they are part of and the academic community as a whole [1].

Unlike students of other educational levels (e.g. undergraduate, master's) [2,3] doctoral students represent some of the most significant intellectual capital of their countries, as they acquire the highest levels of education in their fields of study during this training process, that position them as the future knowledge workers of society [4]. They must manage the intellectual, emotional, technical, and practical demands of academic work to complete their research successfully [2]. Consequently, mental health issues among doctoral students are also associated with impairments in academic performance [4]. This can result in economic and social losses, as mental health challenges may lead to negative consequences for the scientific and technological development of their fields and for the practical areas where this knowledge is applied [4].

Among the factors impacting the mental health and academic performance of this population, the relationship with their advisors stands out [5,6]. Doctoral students and their advisors develop a hierarchical relationship in which the advisor assumes the role of leader [7]. In good conditions, this relationship fosters engagement [7] and can directly contribute to the academic performance of doctoral students. However, incompatibilities between them can negatively affect the quality of this relationship, potentially leading to decreased motivation, workplace conflicts, and mental health issues for students [[7], [8], [9]].

Moreover, the organizational context is also associated with the mental health quality of doctoral students [10]. Graduate programs with unclear norms, expectations, and guidelines are linked to poorer well-being and academic performance outcomes for this population [11]. A lack of clarity can create ambiguous communication between the organization and students, leaving them uncertain about the paths and objectives they should follow during their academic journey. As a result, doctoral students might experience insecurity, anxiety, depression and burnout, all of which negatively affect both their mental health and academic performance [11]. As they are subject to the norms, demands, values and priorities of universities and funding agencies, it may sometimes conflict with their own personal values and priorities and such dissonance triggers doctoral students to significant psychological costs [12].

This review is needed to gather and summarise the evidence of the factors of this dyad’s relationship, as well as the context in which doctoral students are. Therefore, this scoping review aims to identify the relevant factors that have been studied in the relationship and supervision process between advisors and doctoral students that are associated with students’ mental health and academic performance. Additionally, it seeks to analyse if and how the organizational culture of graduate programs has been understood within this context, regarding these outcomes of doctoral students.

## Description of protocol

Despite the relevance of the topic presented in this protocol, previous literature reviews related to the variables of interest in this protocol have focused on understanding the mental health of doctoral students in connection with factors in the academic context [4,12,13] and on key aspects for improving the professional development of supervisors from the perspective of doctoral students [14]. These studies identified the advisor-student relationship as one of the factors associated with the mental health of doctoral students. However, the understanding of this finding does not appear to encompass the multiple facets of this relationship, as it was not the primary focus of the mentioned studies and therefore may have received limited attention in their discussions. Among these review studies, the organizational culture of graduate programs — also referred to as “university processes” and characterized by elements such as unwritten rules, lack of transparency, unclear expectations, and closed decision-making processes—was mentioned in one of them as a source of stress for doctoral students [4]. The authors note that these processes can represent issues within departmental culture, leading to poor communication, mismatched expectations, ambiguities in decision-making during the doctoral process, and limited opportunities for student participation in these decisions. These factors can exacerbate the anxiety experienced by students [4]. Nonetheless, despite the inclusion of organizational culture in this literature review study as a potentially stressful factor for the mental health of doctoral students, the “university processes” overshadow aspects of the advisor-advisee relationship [4]. This highlights the need for further investigations into these variables to better understand how they can be redesigned to achieve greater levels of clarity and openness for students. We suggest that such research could begin with a literature review that directly aims to address this objective, as this protocol intends to do. Thus, it is possible to expand the understanding of mental health and academic performance of doctoral students not only from the dyadic advisor–advisee relationship, but also associated with the organizational culture of doctoral programs.

This proposal is innovative in highlighting the need to understand the interactions between these variables at different levels of analysis: the individual level — based on doctoral students’ perceptions — and the organizational level — focusing on factors related to the organizational culture of doctoral programs. This broadens the understanding that students may be affected in their outcomes by various factors and helps to avoid attributing their mental health issues or academic performance solely to them, thereby preventing a kind of “individual blame.” It recognizes that the conditions to which they are subjected during their doctoral work may also have an impact. This approach may enable future practical contributions, such as the development of strategies and policies aimed at minimizing these impacts, especially considering that impairments to doctoral students’ mental health and academic performance also result in negative consequences for the universities’ own interests and outcomes.

Based on the gaps identified, a preliminary search of Medline (Ovid) was conducted in October 2024, and no current or ongoing scoping reviews or systematic reviews on this topic were found. Thus, this scoping review aims to identify the relevant factors that have been studied in the relationship and supervision process between advisors and doctoral students that are associated with students’ mental health and academic performance. Additionally, it seeks to analyse if and how the organizational culture of graduate programs has been understood within this context, regarding these outcomes of doctoral students.

Research questions

• What are the factors in the advisor-advisee relationship and supervision process that are related to doctoral students’ mental health and academic performance?
• Has the organizational culture of doctoral programs been considered in studies as a factor related to doctoral students’ mental health and academic performance? How?
• What gaps exist in the studies that have analyzed the relationship between doctoral students and their advisors, as well as the role of doctoral program culture, in the outcomes of doctoral students’ mental health and academic performance?

### Inclusion criteria

*Participants:* Doctoral students

*Concept:* Analyze variables of mental health, academic performance and advisor-advisee relationship.

*Context:* Doctoral programmes.

### Types of sources

This scoping review will consider for inclusion quantitative, qualitative, and mixed methods study designs from studies that were published as doctoral theses, master's dissertations, book chapters, and articles from peer-reviewed journals. Additionally, systematic and scoping reviews will also be considered for inclusion in the proposed scoping review.

## Methods

The proposed scoping review will be conducted in accordance with the JBI methodology for scoping reviews and in line with the Preferred Reporting Items for Systematic Reviews and Meta-Analyses extension for Scoping Reviews (PRISMA-ScR) [15]. In this way, the aim is to systematically examine and synthesize the extent, scope, and characteristics of the available evidence on the presented topic, based on the methodological choices made, which are detailed in the following items. This protocol has been registered in OSF as https://osf.io/4w3gd/.

### Search strategy

The search strategy will aim to locate both published and unpublished primary studies and scoping and systematic reviews. An initial limited search of Medline (Ovid) was undertaken to identify articles on the topic. The text words contained in the titles and abstracts of relevant articles, and the index terms used to describe the articles, were used to develop a full search strategy for Medline (Ovid) (see Appendix I). The search strategy, including all identified keywords and index terms, will be adapted for each included information source. The reference lists of studies included in the review will be screened for additional studies.

Studies published in all languages that use the Roman alphabet will be included. The publication period of the articles will not be restricted for database searches. The databases to be searched include Medline (Ovid), Embase (Ovid), Cochrane Library, Web of Science, ERIC, PubMed, CINAHL (EBSCOhost), PsycInfo (APA).

### Study selection

Following the search, all identified records will be collated and uploaded into the software Covidence [16] and duplicates will be removed. Titles and abstracts of the retrieved studies will then be screened by two independent reviewers for assessment against the inclusion criteria for the review. Potentially relevant studies will be retrieved in full and their citation details imported into the JBI System for the Unified Management, Assessment and Review of Information (JBI SUMARI; JBI, Adelaide, Australia) [17]. The full text of selected citations will be assessed in detail against the inclusion criteria by two independent reviewers. Reasons for exclusion of full-text documents that do not meet the inclusion criteria will be recorded and reported in the scoping review. Any disagreements that arise between the reviewers at each stage of the selection process will be resolved through discussion or with a third reviewer. The results of the search will be reported in full in the final scoping review and presented in a PRISMA flow diagram [18].

### Data extraction

Data will be extracted from studies included in the scoping review by two independent reviewers using a data extraction tool developed by the reviewers. The data extraction process will consist of the following steps: i) alignment between the authors regarding the extraction procedures; and ii) extraction of primary data with allocation of relevant information into an Excel spreadsheet. The data extracted will include specific details about the mental health and academic performance of doctoral students in relation to the relationship they have with their advisors and the organizational culture of the postgraduate programs. Thus, the methods used to find this relationship (instruments, reports, analyses) and the identified factors related to the relationship between these actors (main outcomes), relevant to the review question, will also be highlighted. The data extraction tool used follows the recommendations of the JBI Manual for Evidence Synthesis [19]. A draft of the extraction tool is provided (see Appendix II). Modifications will be detailed in the full scoping review. Initially, the information to be extracted from the studies includes: title, year of study, country of publication, authors, objective, analyzed variables, research design, main results, and research agenda. Any disagreements that arise between the reviewers will be resolved through discussion or with a third reviewer. Authors of studies will be contacted up to two times to request missing or additional data, where required.

### Data analysis and presentation

This scoping review will manage qualitative data. PRISMA-ScR reporting guidelines [15] will be consulted and followed. Transform the extracted data, we will apply a thematic approach, which includes reading qualitative data, developing themes, generating analytic categories, and final revision and synthesis through text and tables. At least two of the authors will participate in this stage, who will organize the data from the scoping review in narrative format, through tables and/or visual representation, including maps or diagrams if necessary. The researchers will read the studies in full, extract data, apply the analysis presented to, and finally synthesize data from the studies. These activities will be accompanied by a third and fourth researcher. In cases of conflict, they will be assessed by a third researcher, and a final decision will be made by consensus between the researchers involved in the study. The procedure adopted aims to present the information of interest clearly, organized, and concisely, in order to answer the research questions that originated this scoping review. The involvement of more than one of the authors in this process minimizes the risks of relevant information being omitted or overlooked and contributes to the formulation of discussions and contributions that the study has to make based on its findings.

## Protocol validation

Previous searches did not identify literature reviews directly addressing the objectives of this study: i) the factors present in the relationship between supervisors and doctoral students associated with students' mental health and academic performance, and ii) the relationship between the organizational culture of doctoral programs and the outcomes of mental health and academic performance among doctoral students. However, the preliminary search did find empirical studies that meet the eligibility criteria defined for the construction of this protocol, such as investigations that explored how doctoral students experience research supervision and its impact on their mental health and wellbeing [12], as well as those that assessed the organizational factors relating to the role of doctoral students that predict mental health status [20]. Similarly, literature review studies were also retrieved that highlighted the factors contributing to stress in doctoral settings and, as a result, found that both the culture of doctoral programs, referred to as “university processes”, and the supervisory relationship are among the factors associated with stress in this population. The identification of these investigations suggests the feasibility of conducting the study proposed in this scoping review protocol, considering how the outcomes of interest to be analyzed may have impacts at both the individual level, for doctoral students, and the organizational level, for doctoral programs.

## Limitations

This protocol will not consider studies that have not been written in the Roman alphabet. Furthermore, the first stage of study selection will be carried out based on the screening of titles and abstracts for each study. It is possible that these criteria may introduce limitations in identifying and selecting studies within the scope of this scoping review. However, analyzing thousands of full texts is impractical; therefore, we prioritized title and abstract screening to enhance the feasibility of the review. The search strategy was carefully designed to prioritize sensitivity while maintaining acceptable precision.

## CRediT authorship contribution statement

**Beatriz Cintra Storti:** Methodology, Writing – original draft, Writing – review & editing. **Jorge Sinval:** Conceptualization, Methodology, Writing – original draft, Writing – review & editing. **Yasmin Lynda Munro:** Methodology. **Francisco J. Medina:** Conceptualization. **Marina Greghi Sticca:** Conceptualization, Project administration, Writing – review & editing.

## Declaration of competing interest

The authors declare that they have no known competing financial interests or personal relationships that could have appeared to influence the work reported in this paper.

## Data Availability

No data was used for the research described in the article.

## Appendix Supplementary materials

Image, application 1
