# Supplementary material for: Advisor-advisee relationship and the organizational culture of doctoral programs on doctoral students’ mental health and academic performance: A scoping review protocol
Source: MethodsX. 2025 Jun 11;15:103433. doi: 10.1016/j.mex.2025.103433 (PMC12268343; doi:10.1016/j.mex.2025.103433)
Supplement: Supplementary file 1 [file mmc1.docx]

**Supplementary material *and/or* additional information [OPTIONAL]**

1. Search conducted on 1946 to February, 24, 2025.

| **Search** | **Query** | **Records retrieved** |
| --- | --- | --- |
| #1 | ((doctura* or PhD or postgraduate* or post-graduate* or "post graduate*") adj3 (student* or researcher* or candidate*)).ab,ti. | 3,908 |
| #2 | (supervis* or mentor*).ab,ti. | 124,454 |
| #3 | 1 and 2 | 486 |
| #4 | Mental Health/ or Efficiency/ | 87,462 |
| #5 | ("mental health" or "mental hygiene" or wellbeing or well-being or "well being" or efficient* or productiv*).ab,ti. | 1,394,005 |
| #6 | 4 or 5 | 1416287 |
| #7 | 3 and 6 | 84 |
| No publication date or language search limits were applied. | |  |

*2. Draft data extraction instrument*

| **Scoping Review Details** | |
| --- | --- |
| Scoping Review title: |  |
| Review objective/s: |  |
| Review question/s: |  |
| **Inclusion/Exclusion Criteria** | |
| Population |  |
| Concept |  |
| Context |  |
| Types of evidence source |  |
| **Evidence source Details and Characteristics** | |
| Citation details (e.g. author/s, date, title, journal, volume, issue, pages) |  |
| Country |  |
| Context |  |
| Participants (details e.g. age/sex and number) |  |
| **Details/Results extracted from source of evidence**  **(in relation to the concept of the scoping review)** | |
| Objective |  |
| Study design |  |
| Results |  |
| Research agenda |  |
